# Bluetongue virus: current perspectives on characterisation and the need to formalise nomenclature in an era of rapidly evolving technology

**DOI:** 10.1099/jgv.0.002330

**Published:** 2026-09-08

**Authors:** Ellen M. de Vries, John R. White, Matthew J. Neave, Stacey E. Lynch, Jackie E. Mahar, Vidya Bhardwaj, Debbie Eagles, Tristan Reid

**Affiliations:** 1 Australian Centre for Disease Preparedness, CSIRO, Geelong, Victoria, Australia

**Keywords:** bluetongue, nomenclature, sequencing, phylogenetic

## Abstract

Bluetongue virus (BTV) is a complex, transboundary arboviral pathogen that circulates globally in ruminant populations. Bluetongue disease is a World Organisation for Animal Health listed disease affecting economically important farmed ruminants. BTV is a dsRNA virus from the *Sedoreoviridae* family that has a segmented genome. Classification of BTV traditionally focussed on antigenic characteristics. More recently, however, Sanger sequencing methods have been adopted for typing based on specific genome segment analysis and targeted whole-genome sequencing (WGS) is being more widely applied. Despite these advances, standardised approaches for analysing genomic sequencing data to better define individual BTV strains are lacking. New perspectives are needed to update and unify the current array of approaches surrounding BTV genetic characterisation nomenclature. A transition from single-segment-based serotyping and genotype descriptors to adopting WGS-derived descriptors that better describe a detected virus rather than an individual segment is required. This change will improve the ability of researchers to understand and communicate virus movement pathways with improved granularity. This review paper aims to condense the extensive literature on BTV characterisation, genomic sequencing and virus transmission from an Australian perspective. Recommendations are made to assist with clarity and uniformity around virus characterisation, including genomic definitions and more standardised BTV nomenclature.

## Introduction

 Nearly 40% of global domestic product is generated through the livestock industry [1, 2]. As such, pathogens that threaten to disrupt the industry have high economic consequences [3–5]. According to the World Organisation for Animal Health (WOAH) over 20% of animal production outputs are lost to various animal diseases [6, 7], of which viruses make up more than 24% depending on livestock species [8, 9]. Viruses that threaten agricultural industries are of high importance globally and their attributes need to be understood in order to inform policy, best farming practices, disease management and outbreak preparedness and response activities [10]. Here we review bluetongue virus (BTV), which is a significant pathogen of ruminant livestock species.

BTV is a segmented dsRNA virus within the *Orbivirus* genus in the *Sedoreoviridae* family, official species name *Orbivirus caerulinguae* [11–13]. The BTV genome is composed of ten genome segments (Table 1). Segments 1, 4 and 9 encode the transcription complex (RNA polymerase, helicase/ATPase and methyltransferase/capase, respectively). Segments 3 and 7 encode viral core proteins VP3 (inner core) and VP7 (outer core). Segments 2 and 6 encode the capsid proteins VP2 and VP5, respectively. Non-structural proteins NS1 (viral protein synthesis, virus morphogenesis), NS2 (virus factories) and NS3 (virus egress) are encoded by segments 5, 8 and 10, respectively. Segments 9 and 10 contain secondary ORFs, encoding additional non-structural proteins NS4 (IFN antagonist, segment 9), NS3/NS3a and putatively NS5 (segment 10 [14–18]).

**Table 1.** Segments for BTV including protein coded for, nt and aa lengths

Segment	Protein	Length nt	Length aa
1	VP1	3,954	1,302
2	VP2	2,926	956
3	VP3	2,770	901
4	VP4	1,981	644
5	NS1	1,769	552
6	VP5	1,638	526
7	VP7	1,156	349
8	NS2	1,124	357
9	VP6/NS4	1,046	329; 79
10	NS3/NS3a/NS5	822	229; 216; 59

There are 24 recognised classical (or typical) serotypes as characterised by their VP2 reactivity in virus neutralisation (VN) assays and phylogenetic clustering of the VP2 [19–21]. Additional atypical serotypes continue to be reported globally [19, 22–28]. BTV is predominantly a non-contagious arbovirus [29] which infects ruminant animals, such as domesticated cattle (*Bos taurus, Bos indicus*), sheep (*Ovis aries*) and goats (*Capra hircus*) as well as other cloven-hoofed animals, such as water buffalo (*B. bubalis*), bighorn sheep (*O. canadensis*), deer (Cervidae) and camelids (Camelidae) [30–33]. Clinical disease can be observed in a range of hosts; however, severe manifestations occur most commonly in domestic sheep [6, 30]. The virus’ main targets are endothelial cells, causing tissue and mucosal damage, which results in weight loss, abortions and, in some cases, a cyanotic blue tongue [11]. Death is a common outcome of clinical bluetongue (BT) disease. Subclinical BT disease is mostly reported in cattle and goats, and thus, these may be considered as reservoirs for the virus [34]. Although uncommon, there have been reports of clinical symptoms in European cattle [11, 35–37], suggesting that the epidemiology of the virus is constantly evolving.

**Fig. 1.**
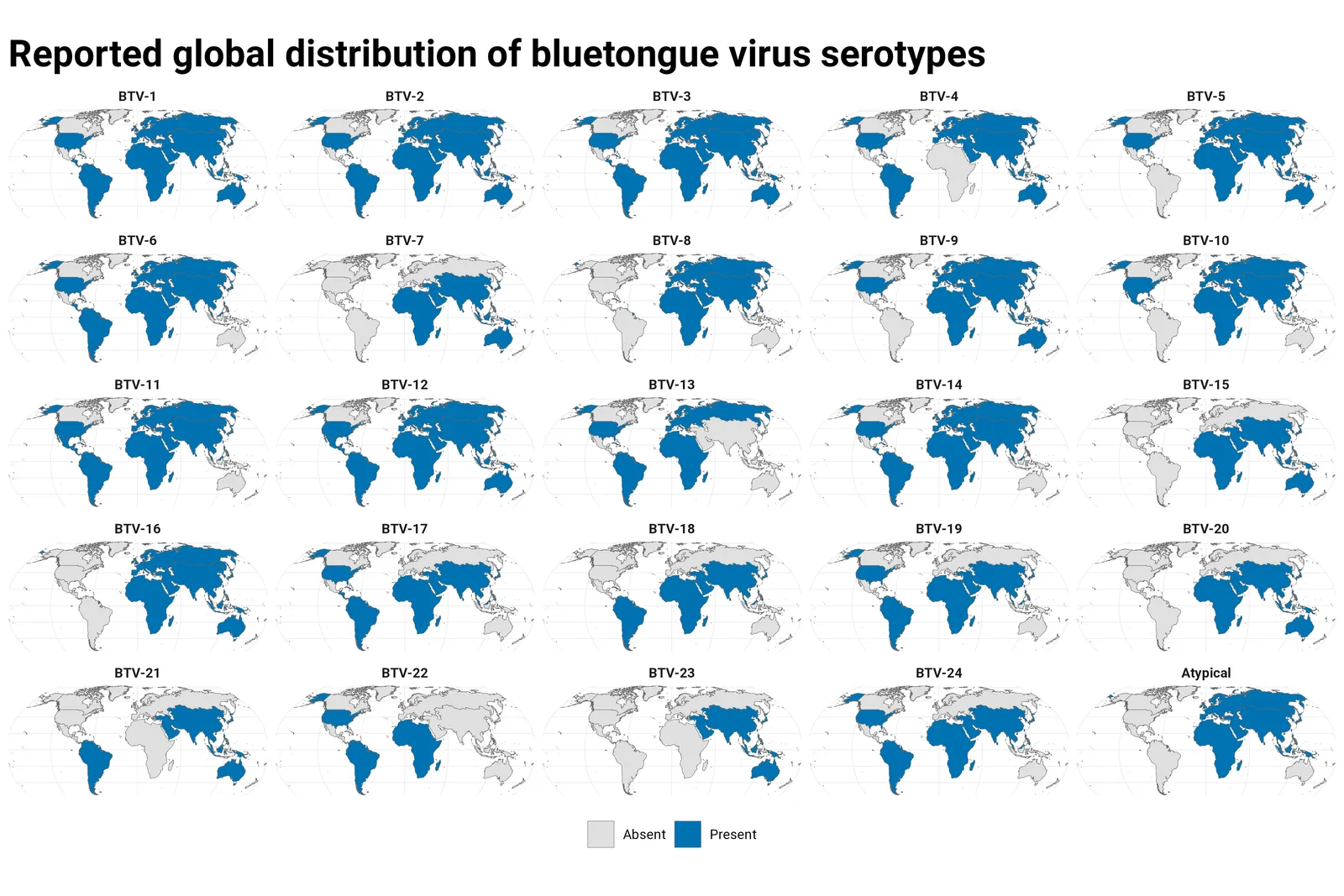
Reported geographical distribution of BTV serotypes globally. Atypical serotypes are represented as a single category.

BTV is transmitted through the bite of competent midge species from the *Culicoides* genus. The distribution of BTV closely mirrors that of competent midge vector species, which are found on all continents except Antarctica and New Zealand (Fig. 1). *Culicoides* midges fall within the Ceratopogonidae family and have been identified as the main vector of BTV. In Australia, the most common vector of BTV is *Culicoides brevitarsis* [38]. Other species including *Culicoides actoni*, *Culicoides dumdumi*, *Culicoides fulvus* and *Culicoides wadai* can all transmit the virus more competently than *C. brevitarsis* but are all less abundant and have a reduced geographical spread when compared to *C. brevitarsis* [38]. This contrasts with Europe, Africa and Asia, where the main vector is *Culicoides imicola* and *Culicoides obsoletus,* both of which are efficient vectors of the virus [39]. The difference in major vector species, combined with differences in serotype spread, may potentially explain why clinical outbreaks are routinely seen in Europe, Asia and Africa, but clinical BT outbreaks have only recently and sporadically been reported in Australia. Current research is focusing on elucidating the roles that different *Culicoides* species play in the pathogenicity and spread of various BTV serotypes [40–42].

This review paper aims to condense the wide literature around characterisation, genomic sequencing and nomenclature of BTV (Table 2), with emphasis given to the situation in Australia. We also aim to highlight current issues with nomenclature and recommend changes to the current system for increased clarity around virus characterisation and communication.

**Table 2.** Schematic representation of current naming systems for BTV with associated references

Naming system	Segment	Major classification	Definition	Reference	Country used
Serotype	2	1–24	Classically based on antibody coagulation of the VP2; most common classification system; recently phylogenetics has been used to determine serotype	Huismans and Erasmus [20] Maan *et al*. [21] Fay *et al*. [155] Erasmus [156]	Global
		25		Schulz *et al*. [28]	
Genotype	3	Malaysia	Partial sequences of the segment 3 are classified as the same genotype if they had <6% nt sequence variation; all genotypes exist under the Australasian topotype of VP3	Pritchard *et al*. [101] White *et al*. [80]	Australia
		Australia			
		Java			
		North America			
		South Africa			
	10	Mediterranean basin, Australia and Asia	Aa-based demarcation; clade distinction based on tree parsimony	Balasuriya *et al*. [123]	America
		Mediterranean basin and South Africa			
		US and Caribbean basin			
		US and Central America			
		Mediterranean basin, South Africa, Caribbean basin and Central America			
	10	Asian group		Pritchard *et al*. [101]	Australia
		US/SA group 1			
		US/SA group 2			
Topotype	Isolate	Eastern	Isolate separation into geographical types that are distinct from each other; most commonly within serotypes and into eastern and western hemisphere origins; based on nt sequence more often than aa; segment % identity for ‘topotyping’ range across segments	Gould [148] Maan *et al*. [157] Boyle *et al*. [69]	Global
		Western			
Nucleotype	2	A – 4, 10, 11, 17, 20, 24	Defined as <35% difference (>66.9% similarity, <61.4% difference) in segment 2 nt sequences across the whole segment; neighbour-joining methods are commonly utilised	Belbis *et al*. [19] Maan *et al*. [21] Boyle *et al*. [69]	Global, commonly India
		B – 3, 13, 16			
		C – 6, 14, 21			
		D – 8, 18, 23			
		E - 5, 9			
		F – 7, 19			
		G – 12, 22			
		H – 1			
		I – 2			
		J - 15			
		K			
		L			
		M		Qin *et al*. [158]	
	6	A – 4, 10, 11, 11, 20, 24	Defined as >76% nt similarity for same topotype	Maan *et al*. [51] Boyle *et al*. [69]	Global
		B – 2, 3, 5, 6, 13, 14, 16, 21			
		C – 1, 2, 9, 23			
		D – 7, 19			
		E – 12, 22			
		F – 15			
		G – 8, 18			
		H - 25			
Genogroup	1	A	P-distance set at 0.2, node threshold set at 75%	Singer *et al*. [154]	Global
		B			
		C			
		D			
		E			
	2	A	Conservatively broken into genogroups with child genotypes; each alignment is based on sequence codons. Representative references for each segment are selected; P-distance is 0.22 with node threshold set at 75% for ClusterPicker	Singer *et al*. [154]	Global
		B			
		C			
		D			
		E			
		F			
		G			
		H			
		I			
		J			
		K			
	3	A	P-distance set at 0.35, 75% threshold	Singer *et al*. [154]	Global
		B			
		C			
		D			
		E			
		F			
	4	A	P-distance set at 0.2, node threshold set at 75%	Singer *et al*. [154]	Global
		B			
		C			
		D			
		E			
		F			
		G			
	5	A	P-distance set at 0.2, node threshold set at 75%	Singer *et al*. [154]	Global
		B			
		C			
		D			
		E			
		F			
		G			
		H			
	6	A	P-distance set at 0.33, threshold set at 75%	Singer *et al*. [154]	Global
		B			
		C			
		D			
		E			
		F			
		G			
	7	A	P-distance set at 0.2, node threshold set at 75%	Singer *et al*. [154]	Global
		B			
		C			
		D			
		E			
		F			
		G			
		H			
		I			
		J			
		K			
	8	A	P-distance set at 0.2, node threshold set at 75%	Singer *et al*. [154]	Global
		B			
		C			
		D			
		E			
	9	A	P-distance set at 0.2, node threshold set at 75%	Singer *et al*. [154]	Global
		B			
		C			
		D			
		E			
		F			
		G			
		H			
	10	A	P-distance set at 0.2, node threshold set at 75%	Singer *et al*. [154]	Global
		B			
		C			
		D			
		E			
		F			

### History of and ongoing emergence of BTV

#### Globally

BT disease has been formally described for more than 120 years, with earlier consistent clinical reports from South Africa dating back to at least 1880 [43, 44]. It no doubt has an archaic ancestry [45], with human colonisation, exotic animal introductions from one country to another and climate change impacts causing a more accelerated spread between biogeographical zones [42]. A full understanding of its origins, diversity and current movement patterns is yet to be elucidated.

There are two popular hypotheses regarding BTV emergence. An initial hypothesis posits that the virus originated in sub-Saharan Africa, where the first records of BTV were reported in 1905 from modern-day Republic of South Africa [39, 44, 46]. The reports mention imported European sheep becoming ill with a fever called ‘Malarial Catarrhal Fever’ but also mention cattle developing symptoms of fever. For affected sheep, a ‘blue tongue’ was identified as a characteristic symptom [47]. Due to the cyanosis of the tongue, the case was not considered to be consistent with other known livestock diseases such as foot and mouth disease. This detection was the first reported outbreak of BT disease [39, 48].

A second origin theory suggests that BTV originated in Asia, most likely the Sino-India region, with subsequent movement throughout western Asia and into Africa via trade routes. Given that domesticated goats originated in the highlands of Iran [49] and have been suggested as the ancestral host of BTV [48], an Asian origin story of BTV would appear logical. India currently has the highest level of BTV diversity, with 22 serotypes reported in the region [50], another strong supporting indicator for Asia as the origin of BTV.

BTV has persisted worldwide in distinct episystem pools. Each pool generally possesses unique ruminant hosts and vector species, and some level of genetically diverging BTV genome constellations due to unique selection pressures [51]. It has been suggested that disease outbreaks primarily occur when viruses spill over from one episystem into another, potentially facilitated by foreign animal introductions or vector incursions [47, 52].

BTV has historically been endemic in Australia (post-ruminant introduction by European colonisers [53]), Africa, Asia and North America [19, 42, 54]. The European BTV system is rapidly changing and appears to be headed towards endemicity of certain serotypes, with BTV detections throughout the Mediterranean, Balkans and Western and Northern European regions in regularity [55].

BT outbreaks have been experienced in the Eastern Mediterranean since the first isolation of BTV-3 in the 1920s [56]. However, at the start of the 2000s, there were several unprecedented incursions of different serotypes causing disease in north and western Europe [54, 57, 58]. The largest outbreak of BT occurred in Western Europe between 2006 and 2009, involving a BTV-8 strain. Countries including Germany, France, the Netherlands and England reported economic losses of upwards of €180 million [5], with over 40,000 livestock cases reported [59]. Although BTV had been seen previously in Europe, this outbreak was identified as a novel introduction, likely originating in Northern Africa, although the specific location is yet to be identified [60, 61]. After apparent eradication, the 2008 outbreak strain re-emerged in 2015, but opinions differ on the origins. Undetected viral amplification in reservoir hosts [62] or the release of contaminated frozen spermatozoa from the original 2008 outbreak released into the then immunonaïve populations have been proposed as potential sources [63]. Regardless, the 2015 reintroduced strain shared near-identical segments to the 2008 circulating BTV-8 strain with only 14 nt changes reported [62, 64]. The 2015 outbreak did not result in such widespread impacts as seen in 2008. The continuing timeline of BTV’s modern global emergence has already been extensively covered [39, 43, 65–68].

#### Australian focus

BTV was first detected in the Northern Territory (NT) in 1975 and confirmed as BTV-20 in 1977 [69, 70]. Serological assays suggest that BTV was well established in northern Australia by this date, which likely began with the introduction of susceptible ruminants during European colonisation of the NT in the mid-1800s [71, 72]. BTV serotypes were detected steadily over the next 50 years in the northern regions of Australia, with confirmatory detections of BTV-21 [73] and BTV-1 [74] in 1979, and BTV-3, 9, 15, 16 and 23 between 1982 and 1986 [74–76]. Since 1986, no new serotypes were detected until the mid-2000s, with BTV-7 reported in 2007 and BTV-2 a year later [77, 78]. Subsequently, BTV-5 and 12 were detected in 2015 [79, 80]. Retrospective testing of archived samples has subsequently revealed that BTV-4 was circulating during this period (between 2006 and 2014) and that the serotype had been present in the country since at least 1995 [81, 82]. No newly established serotypes have since been reported in Australia since 2015.

BTV-1, 16 and 21 are consistently detected year on year across northern and southern extremes of the Australian geographical BTV transmission zone. Areas outside of the transmission zone are defined by having no viral transmission reported for 2 years thus being effectively ‘free’ from BTV [38]. The less commonly isolated serotypes (BTV-2, 3, 4, 5, 7, 9, 12, 15, 20 and 23) are detected sporadically and are normally restricted to the northern zone, with little understanding of the underlying epidemiological factors that contribute to their emergence, persistence and re-emergence. The varying distribution pattern of different *Culicoides* sp. across the continent may be a contributing factor [83].

Despite the presence of BTV, Australia remained free of BT disease until 1989 [84], largely because commercial sheep flocks were outside the most southerly limits of the distribution of *C. brevitarsis* (mid-New South Wales, NSW). In 1989, a BTV-23 virus was detected in a small surveillance flock of Merino sheep near Darwin, NT. This detection of BTV-23 resulted in limited mortalities within the flock suggesting that certain Australian BTV serotypes may have pathogenic tendencies [84, 85]. A limited number of BTV serotypes were confirmed to be circulating in NSW at this time (BTV-1 and 21 [86]) and appeared to possess minimal pathogenicity. Archival testing of sera puts initial incursions of BTV 1 and 21 in NSW far earlier, in the mid-1960s and 1970s (personal communications Peter Kirkland [86]). A small outbreak within a flock of non-commercial NT sheep in 2001 was also reported [87]. No mortalities were reported from this outbreak, although importation and movement of sheep into the NT was subsequently highly regulated [87].

In 2023, two significant but limited BT outbreaks occurred in New South Wales involving serotypes BTV-1 and BTV-16 [88, 89]. The latter serotype was first detected in this state in 2016 [23]. The detection of BTV-1 and 16, associated with potential BT disease, indicated a significant disruption to the previously stable, essentially disease-free status of NSW [90].

The detection of emerging serotypes worldwide is increasing in frequency [91–93], which could be due to anthropogenic changes in climate, changes in land use and subsequent impacts on vector distributions [94–96]. Land changes are less likely to cause new serotype emergence; however, climate change can affect wind patterns, which could influence the direction and frequency of *Culicoides* sp. windborne introduction [97]. In Australia, BTV is hypothesied to be introduced via wind dispersal of *Culicoides* sp. from Indonesia, Timor Leste and possibly Papua New Guinea [98, 99]. This is considered a likely route for introduction of new BTV serotypes into the country, leading to higher virus diversity [69, 98–102].

This area, termed the Northern Australasian episystem, encompassing Timor Leste and Papua New Guinea in addition to the northern tips of Western and Northern Australia and Queensland, displays a viral composition that is vastly different from the Eastern episystem, which extends south from central Queensland into New South Wales. The Eastern episystem is characterised by a more stable system with limited serotype diversity. Although limited in apparent diversity, this system is of great interest given that New South Wales has a large number of domestic sheep and the range of *Culicoides* is slowly extending southwards [38].

Establishment of new serotypes into Australia appears to be uncommon; however, movement of virus genotypes may be a more common occurrence. There is little understanding as to why some serotypes are detected constantly and some are seen sporadically, but whole-genome sequencing (WGS) with high-throughput sequencing (HTS) technologies could shed light on the underlying epidemiological factors that contribute to their emergence, persistence and re-emergence.

#### Current control, surveillance and monitoring efforts

The increase in novel BTV incursions and subsequent rise in clinical outbreaks in the last two decades has prompted many countries to introduce BTV monitoring programmes [50, 103]. The live export industry is most likely to be adversely affected by a BT outbreak. As a major component of the Australian economy, it has a vested interest in a strong surveillance programme, both of endemic species and importations [104]. Currently, there is no globally standardised methodology for BTV surveillance, with different countries employing various surveillance activities to suit their BTV status.

Countries can employ a variety of recommended surveillance activities from the WOAH, which include active vector surveillance [105], antibody testing [105], serological-based surveillance in sentinel herds (including serosurveys) [38, 106] or other molecular-based approaches befitting their country’s BTV status and diagnostic capabilities. Many countries that have endemic BTV do not prioritise BTV surveillance nor incorporate BTV into existing national surveillance programmes, leading to gaps in global knowledge [107].

The laboratory methodologies adopted for BTV characterisation have evolved over time. BTV diagnosis historically relied on serotype-specific VN serological assays. Molecular characterisation techniques such as multiplexed reverse-transcription polymerase chain reaction (RT-PCRs) targeting specific segments (segments 1 and 5 [108, 109], segment 2 [110], segment 3 [111] and segment 10 [112]) as well as ELISAs detecting host response to the virus (competitive-ELISA and sandwich-ELISA VP7 [30, 113–115]) have been described and adopted. As of 2026, no country includes WGS as a formalised aspect of their routine BTV surveillance or characterisation programmes. This is in contrast to current literature proposing that WGS with next generation sequencing (NGS) will play a critical role in understanding the genetic makeup of the viruses, especially during outbreaks [30, 116].

Prior to the advent of HTS technology for WGS, capillary-based sequencing of BTV had two focuses: segment 2 for serotype distinction and segment 3 for topotype designation. This was useful and necessary for understanding the spread of different serotypes globally and informing vaccination strategies. However, due to the segmented nature of the virus genome and the tendency for reassortment, viruses with a common serotype may possess a different genomic backbone, which is missed by sequencing 2/10 segments [31, 91, 117–120]. The cost associated with Sanger and capillary sequencing most likely contributed to the restriction of sequencing, which neither acknowledged nor facilitated the investigation of the potential significance of other segments. With reducing costs of HTS for WGS should facilitate an increase in sequencing data, a trend which can already be seen through NCBI release dates: 247 sequences from 1980 to 2000; 1,239 from 2000 to 2010; 9,538 from 2010 to 2020; and 6,375 from 2020 to 2026.

HTS of BTV is now being adopted to address the limitations of targeted segment sequencing approaches and the high cost of traditional WGS. HTS has already been utilised in European studies as a tool for tracking BTV transmission within countries [34, 121, 122]. The unexpected emergence (2007–2009) and then the re-emergence (2015) of BTV-8 across several European countries demonstrated the utility of this approach. The genome constellation of the 2015 outbreak virus was 99% similar to the 2007 outbreak strain for segments 7, 8 and 9 and had 100% identity with segment 2 [63]. Given the relatedness of both strains as determined by WGS, it was hypothesised that the 2015 BTV-8 was a natural re-emergence of the 2007 BTV-8. By estimating the evolutionary rates of the emerging genomes, Pascall *et al*. [63] showed that the rate of evolution was inconsistent with undetected circulation for 7 years, hypothesising that the 2015 strain was introduced through human influences. The ability to examine viruses beyond their serotype classification and look at the evolutionary pathways of the whole genome is invaluable for understanding novel BTV incursions.

In Australia, monitoring of livestock arboviruses is conducted by the National Arbovirus Monitoring Program [38, 78, 81]. This surveillance programme utilises a combination of vector surveillance and serological surveillance of cattle herds across the country, including regular sampling of sentinel herds and targeted serosurveys during the BTV transmission season. The surveillance outcomes are used to inform the BTV Transmission Zone and support Australia’s trade in agricultural products. The programme produces yearly reports detailing the geographic range of seasonal BTV transmission within the country as well as detections of individual serotypes.

Whilst effective at defining the geographical extent of BTV transmission, there are limitations to serosurveys and ELISA-based surveillance strategies in understanding the patterns of incursion, spread and evolution of BTV within the transmission zone. Until recently, only short-form capillary sequencing was undertaken on a subset of samples to determine the BTV serotype involved in field infections, with additional genetic characterisation activities being limited to partial segment 3 and partial segment 10 genotyping to provide some limited indication of incursions of novel virus strains [94, 101, 123].

#### Identification of genomic determinants involved in expression of BT disease

BTV infection is not always associated with disease and very little is known about the determinants of pathogenicity. There may be many different factors influencing the pathogenicity of the virus, from vector competency to specific genome constellations [124]. Serotype itself is not a singular dictator of pathogenicity [125] and as more serotypes emerge across the globe, a better picture of the pathogenicity of BTV is emerging. Pathogenicity, in the form of clinical BT in field detections, has been reported in eleven out of 24 typical serotypes (BTV-1 [126], BTV-3 [93], BTV-4 [127], BTV-5 [128], BTV-8 [5, 19, 37], BTV-10 [129], BTV-11 [129, 130], BTV-13 [129], BTV-16 [88], BTV-17 [129, 131] and BTV-23 [85]). At the genome level segment 2 ([132]), segment 5 [133]) and segment 10 are hypothesised to play the largest role in controlling pathogenicity [134–137], whereas segments 1, 2, 5 and 6 have been implicated in virulence changes [134–137]. Notably, the majority of pathogenicity work has been undertaken in small animal models rather than larger ruminant species and thus results should be interpreted accordingly.

Whilst progress has been made in potentially understanding the effects of specific segments on pathogenicity, there is still much to be understood. It is particularly complex to pinpoint disease-causing genetic factors in BTV due to the frequency of reassortment during co-infection. A global increase in BTV complete genome sequencing and analysis may clarify the role of each segment in pathogenicity, particularly the complex multi-segment co-contributions – a detail currently being masked when viral segments are examined in isolation [138].

### The use of whole-genome sequencing to understand viral transmission and evolution

Previous research examining the transmission of BTV into and within an Australia context has been conducted [69, 79, 80, 90, 139]. New HTS-based techniques, as well as better bioinformatics and phylogeographic tools, have built on previous observations and added a deeper understanding of the genetic parameters involved in virus spread. Firth *et al*. [90] showed that the distribution of BTV within Australia can be differentiated into two separate zones (Northern and Eastern) upon examination of genotype clusters within all 10 segment phylogenies. The examination of segments at a cluster level, and thus the generation of the total genome constellation, allowed the authors to visualise the geographical movement of and likely reassortment events involving specific segment genotypes. The reassortment of segments between different serotypes may have implications for viral competency (fitness), virus establishment and potential changes in pathogenicity.

The broader use of HTS data will assist in uncovering the current molecular dynamics and historical spread of BTV. The approach of reconstructing historical spread using phylogenetic analysis of sequence information has been widely used for important human and zoonotic viruses [140], but its applications are yet to be fully realised, particularly for animal-specific viruses [141]. Importantly, when considering approaches of phylogenetics for BTV, its proclivity for reassortment should remain in the forefront. Conventional phylogenetic approaches assume that every virus is a direct descendant of another virus, known as ‘identity by descent’ [142], a commonly accepted hypothesis in phylogeny-based analysis [50, 143]. Current works have typically used segment-by-segment analysis to independently examine BTV [48, 50, 121]. This approach assumes that segments may have evolved independently and allows for conventional phylodynamic approaches to be applied. New research is considering the evolutionary impacts that segmented viruses undergo when applying Bayesian phylogeographic models [144] and the validity of these approaches. Previous research has highlighted the interdependency of various segments and suggested that certain segments co-segregate with each other on a preferential basis, especially segments 2 and 6 [119, 139, 143, 145].

#### Nomenclature standardisation for enhanced genome representation

A comprehensive and standardised nomenclature system for classification at a whole-genome level is needed for BTV to achieve consistency in reporting and ease international communication. A new standardised approach must consider both individual segment analysis and the full-genome constellation.

Some segments currently have their own independent naming conventions, including segment 2, which determines serotype; segment 3, which is highly conserved and is used for topotype analyses [146, 147]; and an independent naming convention for segment 10 [123]. In Australia, ‘genotype’ has historically referred to the classification of segment 3 into one of five previously defined groups [101]. The USA, however, classifies segment 10 into ‘genotypes,’ leading to confusion around the term ‘genotype’ for BTV [123]. All segments of the virus can be divided into an ‘eastern’ and ‘western’ lineages, referred to commonly as topotypes, which are distinct groups of viruses defined by their geographic location [148, 149]. Western topotypes are found in Europe, North America and Africa, and eastern topotypes are located in Australia and Asia [146, 150].

Whilst there is no strict standardised approach for naming of sequence data, BTV sequences in genome databases are commonly named by serotype, regardless of segment. This naming convention adds no detail or understanding about the other segments, indicating only that the virus was detected with a specific segment 2 serotype. The reliance of naming non-serotype coding segments on the serotype classification of the virus masks the segment’s own dynamics. This limits the ability to provide nuanced reports on how the virus is changing and moving throughout systems. Understanding the movement of BTV is considered of high importance to many governments and as such an accessible mode of quickly understanding the impact of each segment is critical.

Segment-based naming systems do exist, but they vary geographically, can be confusing and may not have the granularity required to be valuable in some countries. The inconsistency in terminology as well as limited classification systems for segments has prompted us to suggest an overhaul of the nomenclature system for BTV. Furthermore, consideration of reassortment is necessary for understanding how the virus exists and interacts in nature. It has been shown that BTV undergoes a high level of reassortment, reinforcing the importance of genome-constellation-level analysis [50, 139, 143].

A virus nomenclature system similar to that adopted for the avian influenza virus (AIV), or the related orbivirus, *Sedoreoviridae* rotavirus, could function effectively for BTV. These systems note down pathogenic lineages and provide schemes for naming and classifying viruses that incorporate multiple segments; all key aspects that would prove beneficial in a BTV classification system [151, 152].

BTV-GLUE, developed by the Pirbright Institute, the University of Glasgow, the University of Nottingham and PALE-Blu using the GLUE software previously developed [153]. This is a cluster naming system that standardises the naming of BTV segments without referring to the virus’ serotype [154]. This tool allows users to input their own sequence data for phylogenetic classifications of individual segments, described as genogroups. Genogroups are segment-specific groups, and there is no whole virus naming system developed in BTV-GLUE. However, the parameters chosen for genogroup discrimination are very conservative, leading to limitations when examining eastern lineages (based upon our own assessment of several Australian viruses), as there is no clear distinction between Australian, South-East Asian and Indo-Sino viruses, which are classified in the same genogroup.

We propose that BTV-GLUE is modified to achieve a resolution that would allow all countries to easily identify incursions of novel virus segments and track individual segment cluster movements within and between countries. This modified system would be adopted as a fully realised standardised naming system for BTV segment clades.

We further propose that the BTV-GLUE-based segment clade designations form the basis of a genome constellation naming system for BTV, based on a modification of the existing rotavirus or AIV naming systems. This approach has the potential to overcome the limitations and conflicts of current classification systems, enabling rapid identification of significant genomic or epidemiological events and enhancing, simplifying and standardising global communications and reporting of BTV surveillance and BT outbreak response activities. Within this system, we would suggest that the term ‘topotype’ remain solely for purposes of describing the origin of the entire virus (strain), rather than being applied to individual segments.

### Conclusions

The understanding of BTV is ever changing. With the increasing availability of advanced sequencing technologies, the ability to obtain WGS data has become widely accessible. However, genetic characterisation and analysis methods must continue to adapt to take advantage of these new technologies. The utility of WGS for understanding the evolution and spread of BTV is inarguable, but the information derived needs to be easily interpreted and rapidly communicated to effectively inform researchers, governmental and commercial stakeholders alike. The issues highlighted in this review emphasise the need for changes in nomenclature and reporting, to allow researchers globally to communicate their findings and better anticipate and monitor novel and high-impact virus incursions across BTV episystems worldwide.

## Abbreviations

AIV: avian influenza virus
BT: bluetongue
BTV: bluetongue virus
dsRNA: double stranded ribonuclaic acid
ELISA: Enzyme-linkned immunosorbent assay
HTS: high-throughput sequencing
NCBI: National Centre for Biotechnology Information
NGS: Next generation sequencing
NSW: New South Wales
NT: Northern Territory
ORF: Open reading frame
RT-PCR: reverse transcription polymerase chain reaction
USA: United States of America
VN: virus neutralization
WGS: whole-genome sequencing
WOAH: World Organisation for Animal Health
